# XBP1s‐Orchestrated Soluble Mediators: Participants of Oxidative Stress in Renal Ischemia‐Reperfusion Injury Following Donation After Circulatory Death

**DOI:** 10.1155/mi/7037363

**Published:** 2026-05-15

**Authors:** Ji Zhang, Yuanyuan Zhao, Jinbiao Zhong, Handong Ding, Wei Chen, Dongsheng Li, Xianguo Chen, Daofang Zhu, Guiyi Liao, Nianqiao Gong

**Affiliations:** ^1^ Institute of Organ Transplantation, Tongji Hospital, Tongji Medical College, Huazhong University of Science and Technology, Key Laboratory of Organ Transplantation of Ministry of Education, National Health Commission and Chinese Academy of Medical Sciences, Wuhan, 430030, Hubei, China, hust.edu.cn; ^2^ Department of Urology, The First Affiliated Hospital of Anhui Medical University, Institute of Urology and Anhui Province Key Laboratory of Genitourinary Diseases, Anhui Medical University, Hefei, 230022, Anhui, China, ahmu.edu.cn

**Keywords:** donation after circulatory death, ischemia-reperfusion injury, oxidative stress, soluble mediators, XBP1s

## Abstract

Ischemia‐reperfusion injury (IRI) presents an intractable challenge for kidney donors, especially in the era of donation after circulatory death (DCD). Based on in‐depth studies of the renal IRI phenomenon, and the mechanisms involved, an increasing number of soluble mediators are being frequently associated with cellular dysfunction, cell death, and derivative rejection induced by oxidative stress, thus resulting in the failure of DCD kidney transplantation. Many of these soluble mediators are regulated by a spliced form of the X box‐binding protein 1 (XBP1s), a vital effector molecule for endoplasmic reticulum stress (ERS), or exert their functionality by influencing the expression of XBP1s. Owing to the existence of multiple XBP1s‐orchestrated soluble mediators, a variety of biological processes are known to be involved in the occurrence and development of IRI, thus manifesting as profound alterations in DCD kidney transplantation. In this review, we focus on the functionality of these XBP1s‐associated soluble mediators and their roles in oxidative stress following renal IRI. Our goal was to contribute to the advancement of strategies to prevent and treat IRI in the context of DCD kidney transplantation.

## 1. Introduction

Due to the growing disparity between donor kidney supply and demand, the transplant community has been focused on the increasing necessity of grafts from donation after circulatory death (DCD) [1–4]. In 2016, among the 34,854 deceased organ donors reported to the Global Observatory on Organ Donation and Transplantation, the proportion of DCD had risen to 20% [5]. Because DCD donors usually experience a variable period of hypotension, followed by a complete lack of perfusion from the time of circulatory arrest to the start of intravascular cooling [6, 7], DCD donors experience more severe ischemia‐reperfusion injury (IRI) and exhibit increased rates of delayed graft function (DGF) and primary non‐function (PNF) when compared with tissues derived from donation after brain death (DBD) and living donation [6, 8]. Although major advancements in technology, such as machine perfusion [9–12], have been introduced to render tissues more resistant to ischemia or to restrain reperfusion injury, renal IRI is still an inevitable event during the course of organ procurement and transplantation [13–16]. IRI has long been considered a pivotal risk factor for graft dysfunction/nonfunction [2, 17, 18] and acute/chronic rejection [19] through renal cellular injury [20–22], dysregulated immune activation [16, 19], and progressive fibrosis [23], which collectively impact the short‐ and long‐term prognosis of transplantation [24–26]. However, there is still no effective method to prevent and treat IRI; furthermore, the precise mechanisms associated with IRI have yet to be fully elucidated.

**Figure 1:**
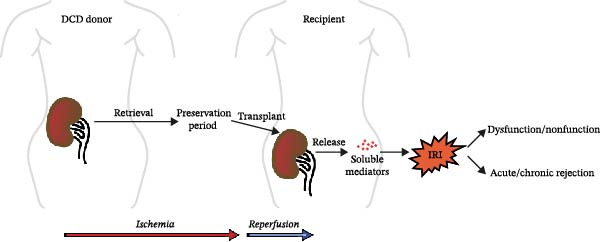
Donation after circulatory death (DCD) kidney transplantation is inevitably accompanied by ischemia‐reperfusion injury (IRI) which promotes the secretion of various soluble mediators from the graft and ultimately impairs the outcome of transplantation.

Oxidative stress is widely recognized as a fundamental contributor to renal IRI [27, 28]. During renal ischemia, the transition from aerobic to anaerobic metabolism results in a decline in cellular pH and a reduced rate of adenosine triphosphate (ATP) production [29]. This metabolic shift leads to the dysfunction of Na^+^/K^+^ ATPases, Na^+^/H^+^ exchangers, and Ca^2+^‐ATPase pumps, thus leading to an increase in intracellular sodium and water, ultimately causing cellular edema [30]. Upon reperfusion, aerobic metabolism is restored, leading to the excessive generation of reactive oxygen species (ROS) that can inflict direct damage on renal cells [26]. Concurrently, both injured and infiltrating cells, such as macrophages, release a range of soluble mediators [31]. These soluble mediators play active roles during the process of IRI in DCD donors [32, 33] (Figure 1). Over the past few decades, many soluble mediators have been found to be released during the aforementioned key pathological processes. This milieu exhibits a dynamic equilibrium among oxidative stress, inflammatory injury, graft rejection, and immune tolerance. For example, while interleukin‐1β (IL‐1β) [34], IL‐6 [35], and tumor necrosis factor‐α (TNF‐α) [36, 37] drive pathogenic inflammation, IL‐10 [38] exerts immunosuppressive and tissue‐reparative effects. Nevertheless, despite such knowledge, there are no commonly accepted therapies for renal IRI in clinical practice. Consequently, there is an urgent need to identify the precise mechanisms underlying IRI and how these mechanisms may relate to these new soluble mediators.

**Figure 2:**
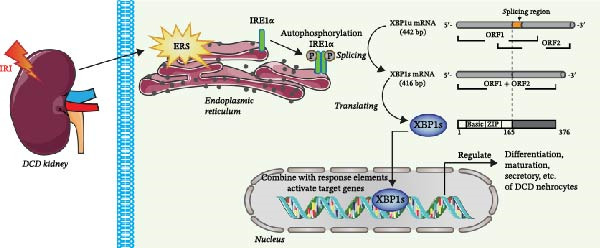
The XBP1 signaling pathway in endoplasmic reticulum stress (ERS) in DCD nephrocytes. IRE1α autophosphorylation activates RNase activity, splices *XBP1u* mRNA, and produces the active transcription factor XBP1s during ERS. XBP1s translocates to the nucleus, where it promotes the transcription of target genes involved in regulating survival, metabolism, and immune modulation.

Previous research has shown that endoplasmic reticulum stress (ERS) during the early stages of IRI after DCD transplantation, along with prolonged machine perfusion, can elicit ERS‐associated gene responses in DCD organs [39–41]. Consequently, in a previous study, we investigated the effects and mechanisms of the spliced form of X box‐binding protein 1 (XBP1s), a vital effector molecule that determines cell fate by alleviating ERS or initiating apoptosis under conditions of irreversible ERS, with regards to the amelioration of IRI [27, 28, 42, 43]. ERS is known to restore protein homeostasis by activating the unfolded protein response (UPR) [42, 44–46]. The UPR comprises three major signaling pathways: protein kinase R‐like endoplasmic reticulum kinase‐eukaryotic translation initiation factor 2α (PERK‐eIF2α), transcription factor 6 (ATF6), and inositol‐requiring enzyme 1α (IRE1α)‐XBP1s [26, 42]. All three branches of the UPR contribute significantly to DCD renal IRI. PERK phosphorylates eIF2α to suppress protein synthesis and promote apoptosis‐related transcription [47, 48]. ATF6 is cleaved in the Golgi by site‐1 and −2 proteases (S1P and S2P), and its active form then translocates to the nucleus to induce chaperone expression, thereby restoring ER homeostasis or triggering programmed cell death [47]. Of the three pathways, the IRE1α‐XBP1s branch exhibits the highest degree of evolutionary conservation [26]. IRE1α autophosphorylation activates endoribonuclease activity to catalyze the excision of an intron from the *XBP1*‐unspliced isoform (*XBP1u*) of mRNA, thereby changing the reading frame to encode a potent transcription factor, XBP1s [26, 42, 49]. As one of the ERS signaling molecules [26, 42], XBP1s participates in cross‐talk between ERS and mitochondrial dysfunction by controlling nuclear factor erythroid 2‐related factor 2 (NRF2)‐mediated ROS signaling. Furthermore, the downregulation of XBP1s has been shown to improve IR‐exacerbated pathological damage and kidney function [26]. Thus, existing evidence indicates that XBP1s is required for differentiation, maturation, and the secretory function of almost all immune cells, including lymphocytes, macrophages, and dendritic cells [42, 50–53] (Figure 2).

Considering the pivotal role of ERS in the regulation of cellular function [42, 44], XBP1s is considered to coordinate or regulate soluble mediators in DCD kidney donors. An increasing number of studies have reported that XBP1s‐orchestrated soluble mediators play a key role in IRI, including mitsugumin‐53 (MG53) [54], CXCL12 [55–57], transforming growth factor‐β1 (TGF‐β1) [58], vascular endothelial growth factor (VEGF) [59], soluble cellular prion protein (sPrP^C^) [60, 61], and soluble biglycan (sBGN) [53, 62]. It is noteworthy that although these mediators may also be subject to regulation by other branches of the UPR, such as PERK or ATF6 [63, 64], the role of XBP1s in orchestrating secretory programs and modulating soluble factors has been more extensively and mechanistically elucidated [50–53].

Herein, we review the specific characteristics of these soluble mediators in renal IRI and their association with XBP1s, thus providing potential targets to develop much‐needed new therapeutic concepts against IRI in DCD renal transplantation.

## 2. Literature Search Strategy

A comprehensive literature search was conducted to identify relevant studies exploring the role of XBP1s and soluble mediators in renal IRI, particularly in the context of DCD. Electronic databases including PubMed, Web of Science, and Google Scholar were queried using combinations of the following keywords: “XBP1s,” “soluble mediators,” “ischemia‐reperfusion injury,” “kidney,” “DCD,” “oxidative stress,” “endoplasmic reticulum stress,” “MG53,” “CXCL12,” “TGF‐β1,” “VEGF,” “ sPrP^C^,” and “soluble biglycan.” The search was not limited to specific years. No minimal sample size and no language restrictions were applied. The reference lists of retrieved articles were also manually screened for additional relevant publications.

### 2.1. MG53

MG53 (also known as TRIM72), a muscle‐enriched member of the tripartite motif (TRIM) family of E3 ubiquitin ligase proteins, is an endogenously secreted myokine that plays a critical role in the repair of cell membranes [65, 66]. MG53 was first discovered in striated muscles [65, 67, 68] and has subsequently been confirmed to be expressed in a variety of different tissue types, including the kidneys and lungs [66, 69]. In addition, MG53 has been detected in the blood stream, tears, and aqueous humor [70].

Previous researchers showed MG53 is a strong membrane repair agent for the treatment of renal IRI [68, 71] and cooperates with myogenic negative regulators, including XBP1s [54]. While generally expressed at low levels in the kidneys, MG53 is significantly enriched in the inner cortex, particularly in the proximal tubular epithelium (PTE), but is not expressed in the medulla [66, 68, 69, 72]. The ablation of MG53 has been shown to limit the self‐repair ability of PTE following exposure to long‐term ischemia‐reperfusion (IR) caused by DCD, thus triggering inflammatory acute kidney injury (AKI) and fibrotic responses [66, 68]. Given that MG53 can cross the glomeruli, previous studies discovered that an intravenous injection of recombinant human MG53 (rhMG53) could sense the oxidized extracellular environment, enter the apical surface of PTE cells, and target injury sites (phosphatidylserine exposed on the outside of the plasma membrane following injury) on PTE cells, thus participating in the assembly of the machinery required for cell membrane repair and preventing the ongoing acute injury to renal epithelial cells induced by IR [66, 68, 72, 73]. Furthermore, MG53 has been shown to enter the nuclei of PTE cells, directly modulate the p65 component of transcription factor NF‐κB, reduce NF‐κB activation, and alleviate kidney fibrosis [72]. Interestingly, XBP1s may cooperate with MG53 in the regulation of NF κB signaling. Both molecules can influence inflammatory and stress responses [42, 72, 73], and it is plausible that they converge on NF κB pathway modulation through direct interaction, coordinated transcriptional regulation, or shared upstream signals. While the precise mechanism of their synergy remains unclear and requires further investigation, this potential collaboration highlights a promising axis for therapeutic intervention. In general, MG53 may play a key role in renoprotection, but it has not been reported in clinical applications, and the specific mechanism of its synergistic effect with XBP1s remains unclear, requiring further research for the prevention of DCD kidney transplant IRI.

### 2.2. CXCL12

The chemokine CXCL12, also referred to as stromal cell‐derived factor‐1 (SDF‐1), was first identified as an essential factor in the bone marrow microenvironment [74, 75]. The function of CXCL12 is to bind to its specific receptors, CXCR4 and CXCR7, which are both expressed on the surface of various stem cells and immune cells [76–78]. Since the discovery of CXCL12, a plethora of research papers have enhanced our understanding of the regulatory mechanisms of CXCL12 in the development of IRI [79–81]. In cultured islets exposed to hypoxia, our research group found that the blockade of CXCL12 downregulated the expression of Myc, upregulated the expression of CHOP and BiP, and finally reduced the protective effect of co‐cultured bone marrow‐derived mesenchymal stem cells (BMSCs) [76].

CXCL12 is expressed in the normal kidney, predominantly by distal tubular cells in the cortex [78]. CXCL12 is highly upregulated in the kidney during DCD‐induced IRI [79] and is secreted into the blood circulation [80], thus reversing the physiological bone marrow‐plasma CXCL12 gradient and recruiting CXCR4^+^ stem and progenitor cells from the bone marrow into the DCD kidney; these cells facilitate the repair of acutely injured DCD kidneys and the treatment of renal fibrosis caused by IRI [78, 81–84]. XBP1s also plays a considerable role in this process, although its influence operates via mechanisms beyond the direct transcriptional control of CXCL12. The expression of XBP1s is known to be deficient in some types of stem and progenitor cells, including mouse embryonic fibroblasts (MEFs), thus resulting in the reduced phosphorylation of extracellular regulated protein kinases (ERK) 1/2 in cells [55, 57], thus abolishing the chemoprotective effect of CXCL12‐CXCR4 signaling axis [55, 57]. It is noteworthy that stem cells with enhanced levels of CXCR4^+^ expression have the ability to prevent oxidative damage by promoting NRF2 signaling and the resultant upregulation of anti‐oxidative response element (ARE) protein [85]. While XBP1s facilitates the migration of CXCR4^+^ stem and progenitor cells to the DCD kidney, it has also been demonstrated that CXCL12 can modulate renal IRI. Researchers have also found that CXCL12 can facilitate mitochondrial metabolism and glycolysis to accelerate repair after renal IRI [79, 80].

In addition to the renal protective effect of CXCL12, it must be acknowledged that the role of CXCL12 in kidney transplantation is multifaceted, exerting both beneficial and detrimental effects on graft outcomes. Elevated levels of CXCL12 can exacerbate chronic allograft nephropathy (CAN) by driving inflammatory infiltration, epithelial–mesenchymal transition (EMT), and progressive fibrosis [48]. CXCL12 also participates in AKI through induced neutrophil infiltration, B‐cell recruitment, and macrophage activation [86–88]. This functional duality is influenced by factors such as expression dynamics, genetic background, and the local microenvironment [48]. The relationship between CXCL12 and DCD kidney deserves more exploration.

### 2.3. TGF‐β1

TGF‐β1 is known to act as a central mediator in the pathogenesis of renal fibrosis [89–91]. Following the onset of IR, resident renal macrophages and renal parenchymal cells secrete the pro‐oxidant TGF‐β1 [92–94] which subsequently increases the activity of NOX2 and NOX4 homologs in the NADPH oxidase family, thus driving ROS formation [95]. NADPH oxidase‐generated ROS is known to contribute to renal fibrosis by stimulating the production of extracellular matrix (ECM) and inducing the transformation of tubular epithelial cells (TECs) to myofibroblasts via EMT [95, 96]. In addition, TGF‐β1‐induced renal fibrosis primarily depends on the TGF‐β1/Smad signaling pathway [23, 94, 97]. TNF receptor‐associated protein 1 (TRAP1), also known as heat shock protein (HSP) 75, is a member of the HSP90 chaperone family that resides principally in the mitochondria [98, 99], and is associated with the TGF‐β/Smad signal transduction pathway [100]. XBP1s acts directly upstream of TRAP1; furthermore, the XBP1s‐TRAP1 axis can inhibit the production of TGF‐β1, prolong G2/M cell cycle arrest, reduce the expression of profibrotic factors, and ameliorate the progression of renal fibrosis during renal IRI [58]. Thus, while TGF‐β1 itself may be influenced by multiple upstream signals, XBP1s exerts a critical inhibitory effect on TGF‐β1‐driven fibrogenesis through a well‐defined molecular mechanism involving TRAP1.

Opinions relating to the specific role that TGF‐β1 plays in apoptosis during renal IRI remain highly debatable. Some data support the fact that TGF‐β1 protects TECs against IR‐induced apoptosis via Smad2 signaling pathways [101]. Guan et al. [92] reported that TGF‐β1 upregulated the expression of anti‐apoptosis protein B‐cell lymphoma‐2 (Bcl‐2) in TECs while protecting cells from TNF‐α‐mediated apoptosis. In other studies, TGF‐β1 was shown to increase apoptosis in the proximal tubules *via* cellular ROS induced by NADPH oxidase [102]. Furthermore, the reduction of TGF‐β1 expression was found to attenuate apoptosis in renal TECs, thus protecting renal cells against IRI [103]. The association between TGF‐β1 and ROS in the pathogenesis of kidney injury and fibrosis has been confirmed. However, the corresponding role of ROS derived from specific NADPH oxidase awaits further investigation. Elucidating the conditions that tilt TGF‐β1 towards repair rather than apoptosis may provide novel therapeutic insights for mitigating fibrotic progression in kidney transplantation.

### 2.4. VEGF

VEGF has been shown to play a central role in vasculogenesis and angiogenesis during IRI [104–106] and binds to the VEGFR to stimulate endothelial nitric oxide synthase (eNOS) phosphorylation, thereby inducing the production of nitric oxide (NO) by the insulin receptor substrate‐1/phosphoinositide 3‐kinase/AKT (IRS‐1/PI3K/AKT) and ERK pathways. This process causes vasodilation, regulates blood flow and pressure, and plays a critical role in the preservation and repair of endothelial glomerular cells and peritubular capillaries [107, 108]. Additionally, NO can effectively attenuate the progression of inflammation and increase the risk of developing chronic kidney disease (CKD) following DCD kidney transplantation [109–111]. Although IRI can increase the expression of *VEGF* mRNA and protein in a variety of cell types and organs in a HIF1α‐dependent manner [105, 112, 113], VEGF levels were shown to be reduced in various renal IR models and DCD kidneys following IR [110, 112, 114]. Kanellis et al. [109] described the redistribution of cytoplasmic VEGF and the enhancement of VEGF receptors (VEGFRs) in the kidneys after IRI; however, these changes were not sufficient to compensate for the lack of VEGF [112].

Therefore, current research is focusing on VEGF supplementation therapy for renal IRI. Mesenchymal stem cells (MSCs) have been shown to promote regeneration and protect the kidneys from IRI via their paracrine or endocrine abilities [115, 116]. VEGF is a crucial mediator of the early and late phases of renoprotective action after IRI in the context of MSC treatment [117–119]. Other studies found that the exogenous administration of MSC‐derived extracellular vesicles (EVs) could effectively alleviate the damage caused by IRI by delivering VEGF directly to renal TECs and increasing the levels of VEGF [118]. Other researchers are investigating novel molecules that can upregulate the levels of HIF1α and VEGF in IRI kidneys via the HIF1α/VEGF axis [104, 106]. Critically, XBP1s contributes directly to this regulatory network through the specific transcriptional control of VEGF. As a transcription factor, XBP1s binds to two regions on the VEGF promoter; these actions have been proven to form a novel upstream regulatory pathway for vasculogenesis and angiogenesis via the transcription of VEGF [59], thus implying that the kidneys are protected against IRI via the regulatory activity of the XBP1/VEGF axis. Consequently, VEGF upregulation therapy may hold promise for the future.

### 2.5. sPrP^C^

The cellular prion protein (PrP^C^), expressed predominantly in the cerebellum, obex, and spinal cord, is a copper‐binding glycoprotein tethered to the cell surface by a glycophosphatidylinositol (GPI) anchor [60, 120]. The infective and misfolded form of PrP^C^ (sPrP^C^) is well‐known as a pathogenic agent responsible for prion diseases [60, 61, 121]. Recent studies determined that PrP^C^ was also expressed in renal tissues, including the proximal convoluted tubules, medullary collecting ducts, extraglomerular mesangial cells (EMCs), podocytes, and endothelial cells [120, 122]. Furthermore, the sPrP^C^ isoform can be secreted into urine by renal epithelial cells [123]. IR‐enhanced endogenous PrP^C^ is a pluripotent protein that is capable of interacting with a diverse range of proteins, including ERK1/2, PI3K/AKT, and cyclic AMP/protein kinase A (cAMP/PKA) [68, 124, 125], and can initiate multiple downstream cellular signaling pathways [126–130]. Following renal IRI, the levels of PrP^C^ are known to be significantly elevated [60]. In PrP^C^‐knockout mice, IRI leads to aggravated tubular damage, enhanced levels of markers for oxidative stress, and more pronounced dysfunction in the mitochondrial respiratory chain [60]. Furthermore, PrP^C^ has been shown to counteract oxidative stress to protect the kidneys via its effects on mitochondria and the ERK1/2 signaling during the early period after renal IRI [60].

Renal IRI is a condition that is known to be implicated with ERS and its adaptive response, notably activation of the IRE1α‐XBP1s pathway which reflects the degree of ERS and plays dual roles in IR‐induced tissue injury [47, 58]. There is a growing body of evidence that suggests that XBP1s can exert a protective effect, while the over‐activity of XBP1s can exacerbate acute cellular damage, reduce the extent of renal fibrosis, and the decline of functionality in transplanted kidneys [26, 58]. Remarkably, the increased expression of renal PrP^C^ relies on XBP1s during IRI [61], thus suggesting that XBP1s/PrP^C^ may represent an axis that can exert nephroprotective effects against IRI. Further reinforcing this regulatory relationship, IRI‐induced concentrations of sPrP^C^ are particularly relevant for the urinary XBP1s transcript levels [61]. This correlation not only supports the role of XBP1s in modulating PrP^C^ expression and cleavage but also positions sPrPC as a promising non‐invasive biomarker for the early detection of renal IRI and for predicting the outcomes of DCD kidney transplantation.

### 2.6. sBGN

Biglycan, a member of the class I family of small leucine‐rich proteoglycans (SLRPs), is a ubiquitous proteoglycan with high homology to decorin [131–133]. BGN possesses a 42 kDa protein core containing leucine‐rich repeats (LRRs), linked to two glycosaminoglycan side chains [132, 134, 135], which interacts with various components of the ECM, thus facilitating the organization and stabilization of the matrix [132, 136, 137]. BGN was previously regarded as an inert ECM derivate; however, recent evidence now indicates that the soluble form of biglycan (sBGN), a bioactive molecule released into the circulation from the matrix via partial proteolysis in response to tissue injury, can bind and induce signaling via different receptors [132, 138].

Upon IRI during DCD kidney transplantation, sBGN can stimulate Toll‐like receptors (TLR) expressed on infiltrating inflammatory cells to secrete cytokines/chemokines (mainly IL‐1β, but also IL‐6, TNF‐α, CXCL1, (C–C motif) ligand (CCL) 5 and CCL2) and exacerbate the influx of immune cells and renal damage [138, 139]. Moreover, cytokines, such as IL‐1β, IL‐6, and TGF‐β, also induce the production of sBGN in immune and renal cells, thus amplifying the action of sBGN as a danger mediator [138, 140–142]. Mechanistically, the NOX2‐mediated production of ROS can impair IL‐1β expression and maturation [143]. sBGN has been shown to engage TLR‐2 and initiate the expression of HSP70 which binds and downregulates the stability of NOX2 by facilitating ubiquitination and proteasomal degradation, thus preventing the NOX2‐mediated generation of ROS [138, 139]. Conversely, NOX1/4‐mediated ROS is known to be beneficial to the expression and maturation of IL‐1β [144, 145]. sBGN enhances ROS production via the TLR/NOX4 signaling pathway [138]. Furthermore, by cross‐linking purinergic P2X4/P2X7 receptors with TLR‐2/4, sBGN can activate the NLRP3 inflammasome, stimulate pro‐IL‐1β mRNA, and upregulate NLRP3/cysteinyl aspartate specific proteinase (caspase)‐1 in a ROS‐ and HSP90‐dependent manner, thus exacerbating the caspase‐1‐mediated maturation and secretion of IL‐1β [138, 139, 146]. In addition, sBGN can selectively interact with TLR‐4 to induce the synthesis of *Nox2* mRNA via the TLR‐4/Toll/IL‐1R domain‐containing adaptor inducing IFN‐β (TRIF) to activate NOX2 in a manner that is dependent on TLR‐4/myeloid differentiation primary response protein (MyD88), thus rescuing the enhanced renal production of IL‐1β [138, 139]. Importantly, research has demonstrated that XBP1s are required for the sustained production of TLR‐activated pro‐inflammatory cytokines by macrophages and dendritic cells. By itself, XBP1s is not sufficient to trigger the significant production of pro‐inflammatory cytokines in infiltrating inflammatory cells, including IL‐6 or TNF‐α. However, XBP1s can be recruited to the promoters of these cytokines, thus synergizing with sBGN‐TLR signaling to activate the expression of inflammatory factors [53, 62]. This synergy potently enhances the transcriptional activation of inflammatory mediators, positioning XBP1s as a critical co‐regulator within sBGN‐mediated innate immune responses.

Thus, sBGN influences the synthesis and activation of danger molecules, including NOX and NLRP3 via different TLR pathways in IRI during the transplantation of DCD kidneys; this represents an insult to the immune system and the regulation of inflammation severity in a rapid manner. This information provides a rational basis for considering sBGN as a promising biomarker for inflammatory damage during renal IRI. By selectively inhibiting the interaction between sBGN and different TLRs, it may be possible to develop a therapeutic option that could reduce deleterious sBGN‐TLR‐mediated inflammation during DCD kidney transplantation.

## 3. Future Perspectives and Considerations

The biological processes underlying renal IRI in DCD donors are highly intricate. Although oxidative stress is commonly acknowledged as a major contributor, current treatments and preventive measures that address one pathway alone have yet to be specifically validated in a clinical environment, especially from the perspective of cold storage preservation and machine perfusion. Similarly, traditional soluble factor‐based treatments have yet to be tested during renal IRI in DCD donors. Although research investigations of anti‐IRI strategies using soluble factors are gradually emerging, there is a notable lack of validated and large‐scale clinical applications.

Although previous researchers have demonstrated that a large number of soluble mediators are closely associated with IRI during DCD kidney transplantation, only a limited body of research has investigated specific links to the UPR pathway, soluble factors, and oxidative stress. Studies have shown that XBP1s is required for development and maintenance of secretory cells [147]. However, in the field of renal IRI research, there is insufficient research on the relationship between XBP1s and a wider range of soluble mediators. For example, Klotho is an anti‐aging transmembrane protein that can be shed into soluble Klotho to protect cardiorenal functions, while it has only found a close relationship between Klotho and ERS, and further mechanistic research has been lacking [148, 149]. Our experimental findings demonstrate that *XBP1* deficiency attenuates renal IRI by inhibiting activation of the NLRP3 inflammasome, leading to reduced IL‐1β and IL‐18 release [27]. Despite these advances, research on XBP1s‐targeted clinical strategies, including drug development, organ preservation techniques, immunosuppressive regimens, and patient management protocols is currently at an exploratory stage, and the extent of their influence on soluble mediators in IRI kidneys requires further investigation [150, 151]. Consequently, there is a specific need to increase our understanding of the complicated mechanisms underlying renal IRI, especially with regards to the relationship between soluble mediators and oxidative stress‐UPR signaling networks. The acquisition of such information may inspire the development of new clinical trials that incorporate novel soluble factors, adapted doses, and delivery methods designed for targeted and sustained release, and gene transfer, to treat renal IRI. Furthermore, individualized combinations of these factors and mechanisms may increase the therapeutic levels of soluble factors and control the levels of harmful soluble factors as a more effective form of treatment that minimizes the double‐edged effects of a single soluble factor.

XBP1s plays a protective role in renal IRI, not only by inhibiting oxidative stress in the early stage of DCD renal IRI as mentioned above but also by promoting renal repair through CXCL12‐CXCR4 signaling‐mediated chemotaxis [82]. Furthermore, its anti‐inflammatory effect is evidenced by the suppression of inflammation via activation of the sBGN–TLR–4/NOX2 pathway [53, 62]. We observed that when renal IRI induced ERS persists, the increased expression of XBP1s may induce mitochondrial‐ROS (mtROS) to promote the activation of NLRP3 inflammasome, leading to the maturation of pro‐inflammatory cytokines IL‐1β and IL‐18 and accelerating cell death [27]. Therefore, the therapeutic effect of XBP1s gene intervention on DCD kidney by regulating soluble factors remains uncertain. A more comprehensive understanding into the interactions between the various mechanisms influenced by XBP1s is essential to better predict and optimize its clinical efficacy.

## 4. Conclusion

**Figure 3:**
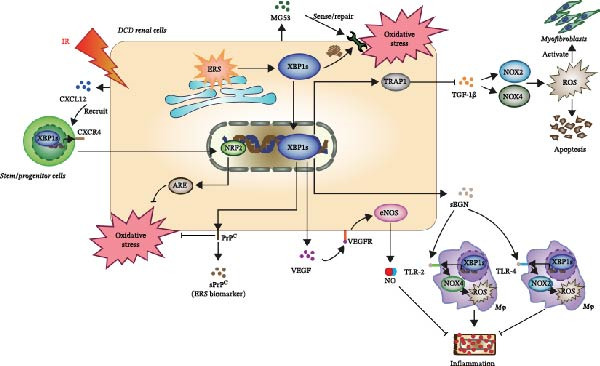
The progression of research showing how XBP1s can influence different soluble mediators in oxidative stress during DCD‐induced renal IRI.

Herein, we describe several XBP1s‐associated soluble factors that have been associated with oxidative stress in renal IRI (Table 1) and summarize how XBP1s may contribute to the active properties of these soluble factors (Figure 3). Collectively, this information should encourage the investigation of other soluble factors to improve the future outcomes of DCD kidney transplantation.

**Table 1:** Soluble mediators in IRI during DCD kidney transplantation.

Soluble mediator	Main cell source	Function in IRI during DCD kidney transplantation	Study types	References
MG53	PTE cells	Participates in cellular membrane repair; attenuates renal fibrosis through suppression of NF‐κB activation.	In vivo (rodent models), in vitro	[62, 68, 69, 72]
CXCL12	Distal tubular cells in the cortex	Facilitates the recruitment of CXCR4^+^ stem and progenitor cells to heal acute damage and fibrosis.	In vivo (rodent and feline models), in vitro	[78, 81–84]
TGF‐β1	Resident renal macrophages; renal parenchymal cells	Aggravates renal fibrosis by stimulating ECM production; transforming TECs to myofibroblasts; plays dual roles in apoptosis.	In vivo (rodent models), in vitro	[95, 96, 102]
VEGF	Podocytes; TECs	Assists endothelial glomerular cells and peritubular capillaries with preservation and repair; resists inflammation.	In vivo (rodent models), in vitro	[107, 108, 117–119]
sPrPC	EMCs; podocytes; endothelial cells	Serves as an indicator of ERS severity and reflects the degree of tissue damage induced by IR.	In vivo (rodent models), in vitro, clinical studies	[60, 61]
sBGN	Almost all damaged cells	Stimulates the expression of TLR‐2/4 on the infiltrating inflammatory cells to regulate secretions of inflammatory factors.	In vivo (rodent models), in vitro	[138–146]

Abbreviations: DCD, donation after circulatory death; ECM, extracellular matrix; EMCs, extraglomerular mesangial cells; ERS, endoplasmic reticulum stress; IRI, ischemia‐reperfusion injury; MG53, mitsugumin‐53; PTE, proximal tubular epithelium; sBGN, soluble biglycan; sPrPC, soluble cellular prion protein; TECs, tubular epithelial cells; TGF‐β1, transforming growth factor‐β1; TLR, Toll‐like receptor; VEGF, vascular endothelial growth factor.

## Author Contributions

Ji Zhang and Nianqiao Gong conceived and drafted the paper. Ji Zhang, Yuanyuan Zhao, and Dongsheng Li prepared the tables and figures. Ji Zhang, Handong Ding, Wei Chen, Xianguo Chen, and Daofang Zhu were involved in reference compilation. Ji Zhang, Guiyi Liao, and Nianqiao Gong revised the manuscript.

## Funding

This research was supported by grants to Nianqiao Gong from the National Natural Science Foundation of China (No. 81873623 and 82170772), the Hubei Chen Xiaoping Science and Technology Development Foundation (No. CXPJJH122001‐2210), the Clinical Research Physician Program of Tongji Medical College, HUST (No. 5001540015), the Zhongsheng Yujin‐Tongji Hospital Cooperation Project (No. 2022044), to Daofang Zhu from the Basic and Clinical Cooperative Research Program of Anhui Medical University (No. 2020xkjT030), to Ji Zhang from the Anhui Hongde‐Shanyi Foundation Clinical Research Development Special Fund (No. KYZX2024025), and the National Natural Science Foundation of China (No. 82400890).

## Disclosure

All authors contributed to the review and approved the submitted version.

## Ethics Statement

No ethics approval was required for this review that did not involve patients or patient data.

## Conflicts of Interest

The authors declare no conflicts of interest.

## Data Availability

No datasets were generated or analyzed during the current study.
